# Effect of Low-Dose Mulberry Fruit Extract on Postprandial Glucose and Insulin Responses: A Randomized Pilot Trial in Individuals with Type 2 Diabetes

**DOI:** 10.3390/nu16142177

**Published:** 2024-07-09

**Authors:** David J. Mela, Marjan Alssema, Harry Hiemstra, Anne-Roos Hoogenraad, Tanvi Kadam

**Affiliations:** 1 Unilever Foods Innovation Centre Wageningen, 6708 WH Wageningen, The Netherlands; 2 Unilever Industries Private Limited, Mumbai 400099, India

**Keywords:** alpha-glucosidase, 1-deoxynojirimycin, glycemic control, starch, rice

## Abstract

Adding mulberry fruit extract (MFE) to carbohydrate-rich meals can reduce postprandial glucose (PPG) and insulin (PPI) responses in healthy individuals. This pilot study assessed the acute postprandial effects of low doses of MFE in individuals with type 2 diabetes. In a randomized cross-over (within-subjects) design, 24 unmedicated adult males and females with type 2 diabetes (mean [SD] age 51.0 [9.3] yr, BMI 27.5 [3.9] kg/m^2^) consumed meals with 0 (control), 0.37, and 0.75 g of MFE added to ~50 g of available carbohydrates from rice. Primary and secondary outcomes were the PPG 2 hr positive incremental area under the curve and the corresponding PPI. Results were reported as mean differences from the control meal with 95% CI. Relative to control, 0.37 and 0.75 g of MFE reduced the mean 2 hr PPG by 8.2% (−20.8 to 6.6%) and 22.4% (−38.6 to −1.9%), respectively, and reduced PPI by 9.6% (−20.7 to 3.0%) and 17.5% (−27.9 to −5.7%). There were no indications of adverse events or gastrointestinal discomfort. MFE additions also led to dose-related reductions in glucose peak and glucose swing. At these levels, MFE appears to dose-dependently reduce acute PPG and PPI in individuals with type 2 diabetes and may be a feasible dietary approach to help attenuate glycemic exposures.

## 1. Introduction

The key treatment target for the management of diabetes is the improvement of glycemic control, which includes not only fasting glucose levels but also postprandial glucose (PPG) responses [1,2]. Drugs that target PPG by slowing the digestion of carbohydrates have been shown to be beneficial for glycemic control, as well as for reducing the risk of diabetes onset in individuals with pre-diabetes [3,4,5]. Dietary guidelines for diabetes include recommendations to help reduce and manage PPG, mainly by controlling the quantity and quality of carbohydrate-containing foods. A possible additional dietary approach is the use of food components that specifically slow the digestion of glycemic carbohydrates in foods and hence the rate of appearance of glucose in blood [6].

Mulberry products are a source of iminosugar 1-deoxynojirimycin (DNJ), which acts as an alpha-glucosidase inhibitor [7]. The inhibition of alpha-glucosidase slows the final step in the digestion of dietary carbohydrates and can thus reduce the rate of uptake and appearance of glucose in blood. A range of naturally-occurring and synthetic iminosugar-based molecules show alpha-glucosidase inhibition, making this the basis for a class of existing drugs (e.g., acarbose and miglitol) and, potentially, also new drugs for managing diabetes [8,9]. We have shown in a series of controlled trials that a well-characterized mulberry fruit extract (MFE) containing 0.5% DNJ reduces both PPG and post-prandial insulin (PPI) responses to test meals in healthy human subjects without diabetes [10,11,12,13]. In those trials, MFE was effective and well-tolerated in tested doses ranging from 0.37 to 1.5 g of MFE (~1.85 to 7.5 mg of DNJ) added to meals containing ~50 g of available carbohydrates from rice or wheat. Importantly, we also confirmed that MFE in this dose range reduces the rate but not the amount of glucose uptake, with no indications of carbohydrate malabsorption or adverse gastrointestinal symptoms [10,11]. 

In contrast, most research testing mulberry products for glycemic control have used mulberry leaf extract (MLE) with 2–10 times higher extract doses and DNJ contents [14,15]. That includes a small number of studies indicating the potential benefits of mulberry products, mainly MLE, in individuals with pre-diabetes or impaired fasting glucose or glucose tolerance [16]. A number of studies on acute PPG responses in those populations have reported some indication of efficacy for PPG lowering using MLE doses from 0.4 to over 3 g, containing (where reported) 6 to 25 mg of DNJ [17,18,19,20,21], although one study found no significant effect at these levels [22]. Most of these trials did not identify adverse effects, although significantly increased breath hydrogen was reported by Mudra et al. (1 g of MLE, DNJ not specified) and Nakamura et al. (3.3 g of MLE, 25 mg of DNJ) [19,20]. 

The present study was intended as a pilot test to assess the potential effects of low doses of a well-characterized MFE on acute PPG and PPI responses in individuals diagnosed with type II diabetes and not on glucose-lowering medications. MFE at the two lowest effective dose levels (0.37 and 0.75 g of MFE containing ~2–4 mg of DNJ) in our previous research [10] were added to ~50 g of available carbohydrates from white rice and compared to the rice alone with no MFE (control). 

## 2. Materials and Methods

### 2.1. General

This was a randomized, double-blind, multi-center, 3-period-balanced order cross-over (within-subject) trial testing two doses of MFE added to boiled rice, compared to rice alone (control), in individuals with type 2 diabetes. Rice was used as a test food because it is a major carbohydrate staple in this population, and it allowed for results to be directly compared to our prior research using MFE added to rice in subjects without diabetes [10]. The pre-registered primary outcome was the effect of the MFE additions on venous PPG, expressed as the percent difference in the positive incremental area under the curve over 2 hr (+iAUC_2hr_), relative to control. Secondary outcomes were the corresponding effects on the PPI total area under the curve (tAUC_2hr_) and the measures of safety and tolerance. Exploratory outcomes were the 3 and 4 hr PPG (+iAUC_3hr_ and +iAUC_4hr_) and PPI (tAUC_3hr_, tAUC_4hr_) responses, the peak glucose level (C_max_), the glucose swing (amplitude of response, defined as the maximum–minimum postprandial concentration [C_max_–C_min_]), and changes in mean urine glucose levels over 4 hr following the consumption of the rice meals.

The study was not planned for formal statistical hypothesis testing (testing for statistical significance), although inferences may be made from the reported means and 95% confidence intervals corrected for multiple comparisons. A formal power (sample size) calculation was not possible because essential information, such as variability in glucose AUC in the intended study population, was not available. For typical pilot/exploratory studies, a minimal number of 12 subjects is recommended as a ‘rule of thumb’, based on feasibility and the diminishing gains in precision with greater sample sizes [23]. A 15% reduction relative to the control was considered a priori to be a desired and physiologically meaningful target effect size. That benchmark roughly corresponded to the 15 unit difference between cut-offs for ‘low’ (55) and ‘high’ (70) glycemic index values, which is associated with health benefits in healthy populations, as well as in individuals with diabetes [24,25]. The study was planned for a sample size of 24 subjects, as we anticipated a larger variability of the glucose and insulin profiles within this population with diabetes, relative to our previous research in individuals without diabetes. 

The trial was prospectively registered at clinicaltrials.gov with the identifier NCT02256332. The clinical phase was executed between 16 February and 16 July 2015 at three study sites in India: Ashirwad Hospital and Research Centre, Ulhasnagar, Thane (site 1); Bangalore Clinisearch, Bangalore (site 2); and Therapeutic Drug Monitoring Laboratory, Mumbai (site 3). Lambda Therapeutics Research Ltd. (LTRL), Ahmadabad, India acted as the central laboratory and data management center. The sponsoring company provided study materials but had no role in participant contact, study execution, or outcome measurement and recording.

### 2.2. Ethical Approval

The trial was conducted in compliance with the Declaration of Helsinki, and the protocol and informed consent forms were approved as Protocol FDS-NAA-1633 by the Ashirwad Ethics Committee (site 1) on 6 November 2014, the Medisys Clinisearch Ethical Review Board (site 2) on 17 January 2015, and the Therapeutic Drug Monitoring Laboratory Institutional Ethics Committee (site 3) on 23 March 2015. 

### 2.3. Participants and Allocation to Treatments

Participants were otherwise healthy adults with type 2 diabetes not treated with drugs. The full in- and exclusion criteria are given in Supplementary Material, Table S1. In brief, eligible individuals were males and females age 20–65, with a BMI of 18–35, with type 2 diabetes (HbA1c ≥ 6.5%) controlled only through diet and exercise, and who were not treated with glucose-lowering drugs in the preceding three months. 

Individuals were provisionally invited to participate by physician investigators at each study site on the basis of medical records and the study inclusion/exclusion criteria. At an initial screening visit, potential participants were given verbal and written explanations and the opportunity to inquire about the details of the research. They were informed of their right to withdraw from the study at any time and gave written informed consent before the start of any protocol-specific procedures. All explanations and procedures were conducted in the native language of participants. Individuals consenting to participate in the study underwent further screening procedures at the study site. This included verification of basic personal and medical history information and a physical examination, including anthropometric measures, urine drug screening, and a blood sample collection for the analysis of HbA1c, hemoglobin, lipids, and routine blood chemistry. Eligibility of the subjects to enter the study was then determined on the basis of the results of screening.

Subjects all received one of each of the test products at test sessions over 3 weeks: control (rice alone, no MFE); rice + 0.37 g of MFE; and rice + 0.75 g of MFE in a balanced-order design. The dose levels were chosen based on efficacy in previous dose-response trials with this same specific extract. With 3 treatment arms (test products), there were 6 possible treatment order sequences. In order to achieve the balanced-order design, equal numbers of the 24 eligible individuals were assigned to one of these six possible treatment sequences using computer-generated random allocations (Supplementary Material, Figure S1). Anyone who dropped out before the first study product administration would be replaced. Anyone dropping out after participating in any of the study product administration periods would not be replaced. The randomization schedule was kept under controlled access by an individual not involved in the study and was unavailable to any personnel who could have an impact on the outcome of the study, e.g., recording of clinical laboratory or other subject data or the collection or evaluation of adverse events. The treatment codes were only broken after the completion of a blind review and a hard lock of the database. 

### 2.4. Source and Characterization of MFE

The MFE (batch No. MF-DC-KQ-111207 Draco Natural Products Inc., San Jose, CA, USA) contained, by weight, 0.5% DNJ (~1.85 and 3.75 mg of DNJ, respectively, in the 0.37 and 0.75 g of MFE dose levels). This is a commercially available aqueous extract produced using a proprietary process and standardized for the DNJ content. The DNJ content and in vitro bioactivity (alpha-glucosidase inhibition), as well as in vivo efficacy of this specific batch of MFE, have previously been confirmed and reported [10,13]. The MFE was packaged in pre-weighed, individually coded aluminum sachets (Pharamivize, Mariakerke, Belgium) containing either 0 (control), 0.37, or 0.75 g of MFE plus mannitol to bring the total weight of the sachet contents to 1 g.

### 2.5. Test Meals

Each test meal consisted of a serving of boiled rice with 0, 0.37, or 0.75 g of MFE added. A single serving of rice was prepared using 64 g of raw Sona Maroori (Sona Masuri) rice containing ~50 g of available carbohydrates, sourced and prepared as previously described [12]. Each portion of rice was prepared in a rice cooker with the addition of 140 mL of water. Sachets containing the control or MFE were then stirred into each serving of prepared rice. Participants and staff serving the rice were blind to the presence or dose of MFE, which, at these levels, has negligible effects on sensory attributes. 

### 2.6. Test Day Procedures and Data Collection

Subjects participated in three test days separated by 5–7 days. They arrived at the test facility at around 18.00 on the evenings prior to test days and stayed at the facility at least 4 hr after the administration of the test meals. Subjects were asked to avoid strenuous physical activity and the consumption of alcohol for at least 24 hr and were fasted for at least 10 hr prior to the start of the test meals. They were given identical, standardized evening meals of a fixed quantity and not permitted to consume any other food or beverages except water, which was allowed up to 1 hr prior to test meals.

On test days, subjects consumed the test meals immediately after preparation, together with 350 mL of water, as a morning meal. The moment the first mouthful of rice was swallowed was recorded as t = 0 (0 hr, 0 min). Subjects were instructed to consume the study product within 15 min. If a subject was not able to finish the preparation within this time frame, the 15 min blood sample was taken, and they continued consuming the study product immediately after blood sampling. Subjects did not consume any other food and were allowed, at a maximum, an additional 500 mL of water until the last blood sample was taken and a gastrointestinal discomfort questionnaire was completed at t = 4 hr. The actual quantity of water consumed by each subject was recorded on their first test day, and it was the same amount permitted on subsequent test days. 

For the analysis of plasma glucose and serum insulin, two consecutive baseline blood samples were collected with a maximum gap of five minutes and within a period of 15 min before the start of the test meal. Following the start of the test meal at t = 0 min, blood samples were collected at t = 15, 30, 45, 60, 90, 120, 150, 180, 210, and 240 min for analyses.

Venous blood samples (6 mL) were collected from a valve on an indwelling cannula placed in the forearm (antecubital vein), which was kept patent by injections of 0.5 mL of normal saline solution. At each timepoint, the first 0.5 mL of blood was collected into a 2 mL syringe and discarded to avoid saline contamination. Blood was subsequently collected into vacuumized sample collection tubes attached with a Luer adapter to the cannula. If the cannula was blocked or there was difficulty in drawing blood through the cannula, blood samples could be taken either directly from the cannula into a 10 mL syringe or by a fresh vein puncture using a 22-gauge needle.

Subjects completed a gastrointestinal discomfort questionnaire 30 min prior to and at 240 min after the consumption of the test meals. The questionnaire asked whether subjects had experienced any flatulence, nausea, bloating, or bowel pain, with each symptom separately rated as none, mild, moderate, or severe.

For the analysis of urine glucose, a baseline urine sample was collected approximately 30–45 min before each test meal. Urine subsequently produced by each subject was collected in pots during the 4 hr following the consumption of test products and immediately refrigerated. The last urine sample was collected at approximately 4 hr after the start of the test meal when other procedures were completed. 

### 2.7. Blood and Urine Sample Handling and Analyses

Duplicate aliquots of plasma (for glucose) and serum (for insulin) were obtained from 2 and 4 mL of blood, respectively. Samples were centrifuged at 18–25 °C within 60 min of collection for serum and within 45 min for plasma at 2500–3000 rpm for 10 min. Proper clot formation was ensured before centrifugation for serum separation. The samples were frozen and stored at −22 ± 5 °C within 15 min of centrifugation. The volumes of individual baseline urine samples and of the pooled urine samples following the consumption of the test products were measured, and two 6 mL aliquots of each were frozen for later analyses. One set of all samples was shipped, frozen, to LTRL for analyses, and a duplicate set was retained at each study site in case of loss or the need for re-analysis. 

Glucose in plasma and urine was analyzed using a Vitros 5,1 FS Chemistry System analyzer (Ortho Clinical Diagnostics, Inc.; Raritan, NJ, USA). Insulin was measured by electrochemiluminescence on a COBAS e411 immunoassay analyzer (Roche; Basel, Switzerland). 

### 2.8. Adverse Event Recording

Adverse events were defined as mild, moderate, or severe, according to the need for treatment and the level of interference with normal daily activities. The possible relationship of any adverse event to the study meals or procedure was defined as unrelated, unlikely, possibly, probably, or definitely related, according to the criteria, including the association with time and the likelihood of alternative explanations.

### 2.9. Statistical Analyses

Statistical analyses were carried out according to a pre-specified plan, and no interim analyses were planned or performed. The primary endpoint was calculated with a linear mixed model using Log(+iAUC_0–120min_) as the response. The models always included baseline, subject_baseline, treatment, and treatment sequence order as predictors. Baseline was the mean baseline value for that visit for that subject; subject_baseline was the mean baseline score over all visits, and it was included to avoid possible bias in the estimates of the treatment effect due to the use of a mixed model and the inclusion of a different baseline value at each visit for the subject. The error term of the model was assumed to be normally distributed. Other predictors, such as body weight, gender, and visit (a categorical variable identifying the number of test day visits), could be included in the model based on statistical relevance. The model-derived estimated differences in treatment effects (obtained on a log scale) were back-transformed into a percent change and its associated confidence interval. 

An analogous statistical model was used for the secondary outcome PPI tAUC_2h_ and the corresponding exploratory results for PPG and PPI over 3 and 4 hr. For these PPG and PPI outcomes and the exploratory endpoint C_max_, the percent change at each dose of MFE was calculated relative to the control rice, with no MFE as a reference. The other exploratory endpoints, glucose swing (C_max_–C_min_) and pre- vs. post-meal glucose concentration in urine, were determined for each treatment and reported as absolute differences for each MFE treatment relative to the control.

This was a pilot study that was not planned or powered for formal statistical hypothesis testing (determination of *p*-values). The relevant results are therefore presented as the size of the estimated treatment effect: mean and 95% confidence intervals (CI) were used as measures of its reliability using a Dunnett correction for multiple comparisons with the control. Results where the 95% CI for the difference between an MFE treatment and control did not include a null effect (zero) would therefore be analogous to *p* < 0.05. 

## 3. Results

### 3.1. Analysis Population

Subject baseline characteristics are shown in Table 1. Of the 24 individuals entering the trial, 22 completed all treatments. One subject provided no usable treatment-related data, and one dropped out after their first test session (see Consolidated Standards of Reporting Trials [CONSORT] subject flow diagram, Supplementary Material Figure S1). During blind review, high baseline values for both plasma and urinary glucose (exceeding 10 mmol/L and 250 ug/mL, respectively) were observed from one subject at all visits and from a further subject at one visit. These values were attributed to subjects not being fasted, and it was judged that the glucose and insulin data from those subjects/visits should be excluded from the statistical analyses. Available data from all other participants and visits were used, and no distinction was made between the intention-to-treat and per-protocol analyses.

### 3.2. Primary and Secondary Outcomes

The primary outcome PPG +iAUC_2hr_ showed dose-related reductions from the addition of MFE (Table 2). The mean relative reduction of 22.4% for the 0.75 g dose of MFE was robust and exceeded the pre-specified target effect size of 15%. PPI tAUC_2hr_ was also reduced in a dose-related way by the addition of MFE (Table 3), with a mean 17.5% reduction for the 0.75 g dose of MFE.

There were minimal indications of any intolerance or safety issues, and none could be specifically attributed to the consumption of MFE. On the gastrointestinal discomfort questionnaire, one subject reported mild bloating at one visit and mild nausea at another, both at t = 240 min. One other subject reported mild bloating at baseline (t = −30 min) at one visit. No other symptoms of gastrointestinal discomfort were reported by any other subjects at any timepoints. In total, five adverse events were recorded for three subjects (one case each of vomiting, dizziness, and nausea and two cases of abdominal distension). All events were judged to be mild, did not require medical treatment, and were unrelated to the specific study products or procedures.

### 3.3. Exploratory Outcomes

The profiles of PPG and PPI responses over the full 4 hr postprandial period are shown in Supplementary Material, Figures S2 and S3. There were limited effects of MFE on the PPG responses when summed over 3 and 4 hr (+iAUC_3hr_ and +iAUC_4hr_), although these were slightly reduced by the higher MFE dose (Supplementary Material, Tables S2 and S3). However, both the 3 and 4 hr PPI responses (tAUC_3hr_ and tAUC_4hr_) were clearly reduced by the higher dose of MFE (Supplementary Material, Tables S4 and S5). 

The addition of MFE produced modest, dose-related reductions in C_max_ (Supplementary Material, Table S6) and more consistent reductions in glucose swing (Supplementary Material, Table S7). Pooled urine glucose levels following the consumption of the test meals were highly variable but, on average, increased similarly from the baseline in all groups (Supplementary Material, Table S8). 

## 4. Discussion

The addition of relatively low doses of MFE to rice led to dose-related reductions in the acute PPG and PPI responses in individuals with type 2 diabetes. This was most apparent during the immediate (2 hr) post-prandial period and was accompanied by apparent reductions in the post-meal peak glucose levels and glucose swing. The effects were more consistent and robust for a dose of 0.75 g of MFE than a 0.37 g dose, and at the higher dose, there were also reductions in PPI over the full 4 hr measurement timeframe. 

The effects seen here were consistent with our previous research on individuals without diabetes, as was the absence of any indications of intolerance or other adverse effects of MFE. The mean reduction in PPG following the higher dose of MFE also exceeded our pre-defined ‘desired’ target reduction of ≥15%. Although we previously reported similar reductions in PPG and PPI with doses of 0.37 g of MFE containing ~1.85 mg of DNJ, that level appears to be close to the lower limit of efficacy [10]. A dose of 0.75 g of MFE may therefore be advised to ensure more reliable effects across different carbohydrate sources and populations [12]. 

DNJ in mulberry extracts has a well-known primary mechanism of action, similar to alpha-glucose-inhibiting drugs, which are effective in the treatment of diabetes and in reducing the risks of co-morbidities. The benchmark 15% change in PPG achieved here corresponds to about one-third of the observed effect on the PPG of drugs (miglitol, acarbose) used to reduce PPG [4]. This seems a reasonable effect size for a dietary adjunct, given that the drugs are available only with a prescription for patients under medical supervision and that gastrointestinal discomfort due to carbohydrate malabsorption is a common side-effect of these medications [26]. Some studies using high doses of MLE have reported increases in breath hydrogen, indicative of carbohydrate malabsorption [19,27]. Thaipitakwong et al. reported that an MLE dose containing 18 mg of DNJ resulted in a high incidence of bloating and flatulence, which was not seen at DNJ levels of 6 or 12 mg [28]. Comparisons between the MFE used here and studies using other mulberry extracts must be made with caution, however, due to differences in (often unreported) DNJ levels and overall product compositions. 

We have found no indications of malabsorption or intolerance here or in studies using MFE at levels up to twice those in the present trial [10,13]. This may be due in part to the relatively low dose of DNJ and its high and rapid absorption in the proximal intestine, thus limiting the period that it is present in the gut lumen, affecting carbohydrate digestion [29]. Ingested DNJ is estimated to reach maximum plasma concentrations in about 30 min, much faster than common alpha-glucosidase-inhibiting drugs (acarbose, miglitol), which have a longer duration of presence and action in the gut [29,30]. The short period of activity of DNJ has implications for effect sizes and for its suitability for use with food, relative to medications. This is advantageous for mitigating potential side effects whilst still moderating the initial and peak glucose responses following meals, but it limits the effects over the longer (3–4 hr) post-prandial period, as observed here. 

We and others mainly attribute the observed effects of mulberry extracts on PPG to the presence of DNJ. Although DNJ can be found in a limited number of other plants or can be synthesized by certain bacteria [29,31], to our knowledge, DNJ is not found in meaningful amounts in any dietary sources other than mulberries. It is, however, also possible that other iminosugars or minor components of MFE could also contribute to these effects. 

While the present results are largely in line with our previous research on MFE and the wider literature on other mulberry extracts, there are a number of limitations to this study. Most mulberry extracts intended for food or supplement use are derived from processing the raw materials with water and ethanol [29]. Although the MFE used here is commercially available and standardized for its DNJ content, the exact production process is proprietary, and MFE derived from other sources may, therefore, differ in their content of other minor components. It is possible that some part of the variation between studies of mulberry extracts may be attributable to these differences in composition. 

This was a fairly small pilot trial, not formally powered for inferential hypothesis testing. Although this was adequate for the current purpose, a larger test population would provide for more confident estimates of effect sizes. Venous blood was collected to limit the burden on subjects. This is unlikely to bias the results in relation to the effects of MFE but may produce lower and more variable glucose values than capillary blood [32]. Therefore, while this should not affect the general conclusions of this pilot trial, other methods of blood collection may allow for a more precise estimate of effect sizes.

Effects of MFE on PPI were a secondary objective, which was mainly to re-confirm that effects on PPG were not attributable to a disproportionate insulin response. The reductions in insulin responses are most likely an indirect result of reducing the rate of glucose uptake and thereby also reducing the stimulation of insulin secretion. However, possible effects on insulin secretion have not been directly tested. In future research, a greater focus on measures of insulin release and action may be advised for this population in particular, as well as considering longer and repeated exposures and markers of sustained glycemic control. 

## 5. Conclusions

This trial adds to a series of studies on mulberry extracts and MFE specifically, supporting their safety and efficacy for reducing acute PPG and PPI responses to common dietary carbohydrate sources and different populations [10,11,12]. Together with the present results, the evidence indicates that a modest dose of 0.75 g of MFE containing 3.75 mg of DNJ consumed with a digestible carbohydrate source is likely to cause mean reductions in the range of about 10–25% in the 2 hr PPG AUC, as well as reduce PPI, peak glucose, and glucose swing in healthy individuals and individuals with diabetes.

## Figures and Tables

**Table 1:** Subject baseline characteristics (N = 24; 11 male, 13 female).

	Mean	SD	Range
Age, yr	51	9.3	34–63
Weight, kg	69.7	20.3	47.6–88.2
BMI kg/m^2^	27.5	5.9	21.0–34.8
HbA1c, %	7.2	0.5	6.5–7.8

**Table 2:** Plasma glucose response over 2 hr following the consumption of mulberry fruit extract (MFE) added to boiled rice.

Intervention	N	Mean Glucose +iAUC_2hr_ (Lower, Upper 95% CI), min·mmol/L	Mean % Difference, MFE vs. Control (Lower, Upper 95% CI)
Control	21	346 (307, 390)	
Control + 0.37 g MFE	22	318 (281, 360)	−8.2 (−20.8, 6.6)
Control + 0.75 g MFE	20	269 (215, 336)	−22.4 (−38.6, −1.9)

**Table 3:** Serum insulin response over 2 hr following the consumption of mulberry fruit extract (MFE) added to boiled rice.

Intervention	N	Mean Insulin tAUC_2hr_ (Lower, Upper 95% CI), min·mIU/L	Mean % Difference, MFE vs. Control (Lower, Upper 95% CI)
Control	21	6470 (5464, 7661)	
Control + 0.37 g MFE	22	5847 (4949, 6908)	−9.6 (−20.7, 3.0)
Control + 0.75 g MFE	20	5334 (4501, 6325)	−17.5 (−27.9, −5.7)

## Data Availability

The datasets are not publicly available, as participants did not give express consent for this. Data described in the manuscript, code book, and analytic code can be made available upon request, pending application and approval.

## Supplementary Materials

The following supporting information can be downloaded at: https://www.mdpi.com/article/10.3390/nu16142177/s1, Table S1: Inclusion and exclusion criteria; Table S2: Plasma glucose area under the curve response over 3 hr; Table S3: Plasma glucose area under the curve response over 4 hr; Table S4: Serum insulin area under the curve response over 3 hr; Table S5: Serum insulin area under the curve response over 4 hr; Table S6: Maximum glucose level (C_max_) over 4 hr; Table S7: Glucose swing (C_max_–C_min_) over 4 hr; Table S8: Pooled urine glucose concentrations over 4 hr; Figure S1: Consolidated Standards of Reporting Trials [CONSORT] subject flow diagram; Figure S2: Plasma glucose response over 4 hr; Figure S3: Serum insulin response over 4 hr.

## Abbreviations

C_max_	Maximum recorded concentration of glucose in the post-prandial period
C_min_	Minimum recorded concentration of glucose in the post-prandial period
DNJ	1-deoxynojirimycin
iAUC	Incremental area under the curve
MFE	Mulberry fruit extract
MLE	Mulberry leaf extract
PPG	Postprandial glucose
PPI	Postprandial insulin
tAUC	Total area under the curve
