# Supplementary material for: Effect of Low-Dose Mulberry Fruit Extract on Postprandial Glucose and Insulin Responses: A Randomized Pilot Trial in Individuals with Type 2 Diabetes
Source: Nutrients. 2024 Jul 9;16(14):2177. doi: 10.3390/nu16142177 (PMC11280168; doi:10.3390/nu16142177)
Supplement: Supplementary file 1 [file nutrients-16-02177-s001.zip › nutrients-3073509-supplementary.pdf]

Mela et al, “Effect of low-dose mulberry fruit extract on postprandial glucose and insulin responses: A randomized pilot trial in individuals with type 2 diabetes”

SUPPLEMENTARY MATERIAL (tables and figures in order of reference in manuscript)

Table S1. Inclusion and exclusion criteria

Subjects who met the following criteria could be included in the study:

- Male and non-pregnant, non-lactating females with type 2 diabetes (confirmed by two independent measurements of HbA1c ≥6.5% but <8.0%), who are either treatment naive (controlled solely through diet and exercise) or have not been treated with glucose lowering drugs for the preceding three months
- Age ≥20 and ≤ 65 years of age
- Body Mass Index (BMI) of ≥18 and ≤35 kg/m^2
- Willing and able to give written consent to participate in the study
- HbA1c confirmed by two measurements as ≥48 mmol/mol (6.5%) and ≤53 mmol/mol (7.0%) or, at the discretion of the study physician, HbA1c >53 mmol/mol (7.0%) but <63 mmol/mol (8.0%)
- For subjects <40 years old, Type 1 diabetes ruled out by the study physician
- For patients with newly diagnosed hyperlipidaemia and not on medication: Lipid values of Total Cholesterol 200-350 mg/dL, LDL 100-170 mg/dL, HDL ≥40 mg/dL (males) or ≥50 mg/dL (females), Triglycerides <199 mg/dL
- Hemoglobin level within clinically acceptable range (males 12-17 gm/dL, females 11-15 gm/dL) as judged by the research physician
- Other routine blood chemistry parameters within normal range
- No medical conditions which might affect study measurement or measurement of HbA1c (e.g. chronic renal disease), as judged by the study physician
- Blood pressure up to 140/90, managed if relevant with lifestyle intervention and medications other than ACE (Angiotensin Converting Enzyme) inhibitors or ARBs (Angiotensin II Receptor Blockers)
- For subjects using other medications: Only medications which do not impact insulin sensitivity
- For subjects with hyperlipidemia: Use of statins if relevant
- Willing to comply to study protocol during the study
- Agreeing to be informed about medically relevant personal test-results by study physician
- Willing to refrain from drinking alcohol for at least one day before the blood withdrawal and study product administration
- Having accessible veins on arms as determined by examination at screening

Exclusion criteria:

- Being an employee of Unilever or Hindustan Lever Ltd. or the study site
- Use of tobacco products
- Consumption of alcoholic drinks daily, or more than 2 drinks in a day, or more than 8 drinks per week
- Participation in any other biomedical study 3 months before screening visit day of this study and/or participating in any other biomedical study during the screening period.
- Reported work in night shifts (between 23.00 and 6.00 hrs)
- Chronic medication other than those allowed in the inclusion criteria
- Reported weight loss/gain ≥10% of body weight in the 6 months preceding screening
- Blood donation for 2 months prior to screening
- Urine analysis indicating drug abuse

- Allergy to any food or cosmetics
- Pregnant or planning pregnancy during the study period
- Lactating or been lactating for 6 weeks before pre-study investigation and/or during the study period
- Intense exercise >10 h/week (defined as exercise which induces sweating and causes sufficient breathlessness to limit conversation)

Figure S1. Consolidated Standards of Reporting Trials (CONSORT) subject flow diagram.

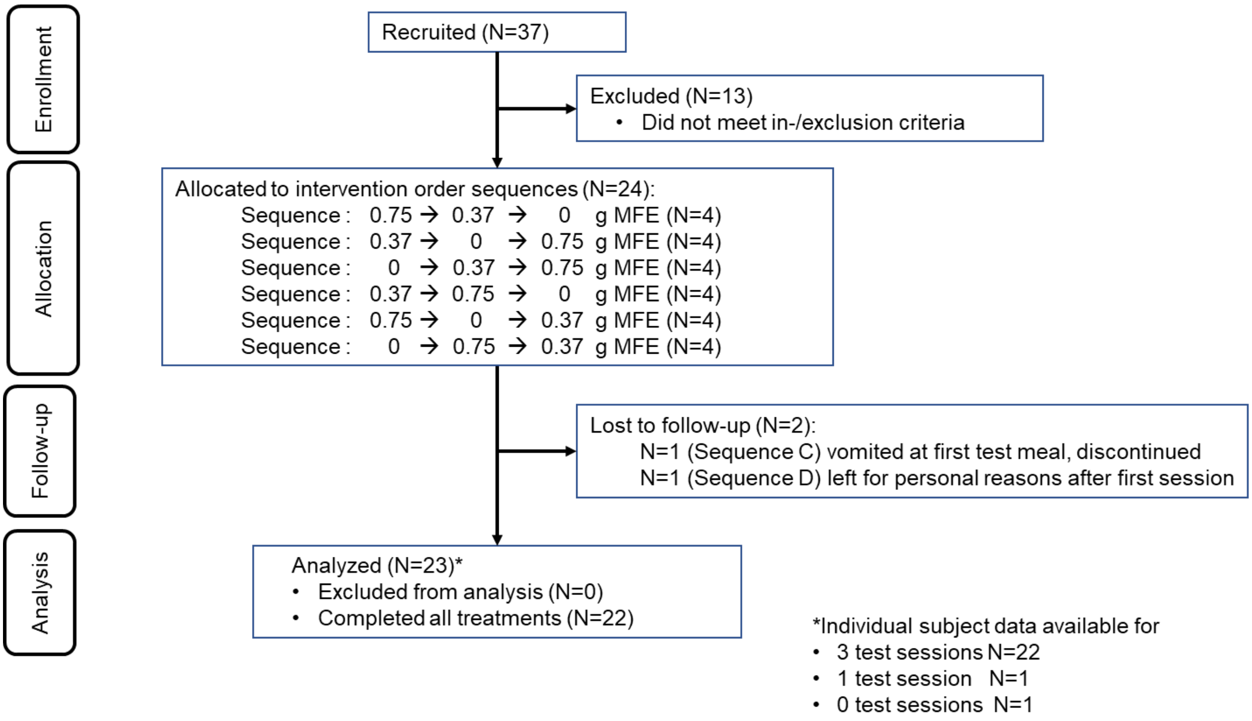

Figure S2. Plasma glucose response over 4 hours following consumption of mulberry fruit extract (MFE) added to boiled rice.

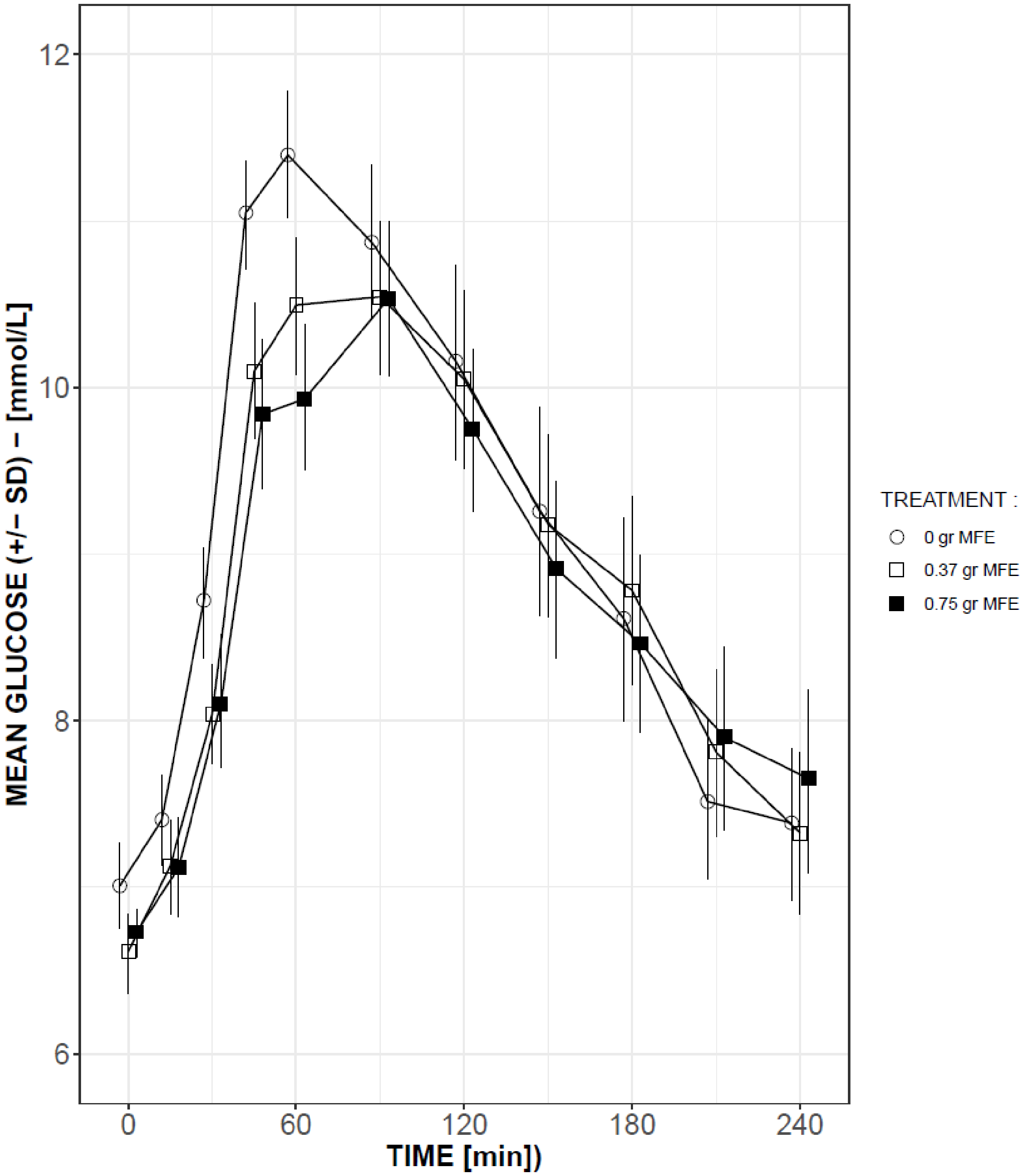

Figure S3. Serum insulin glucose response over 4 hours following consumption of mulberry fruit extract (MFE) added to boiled rice.

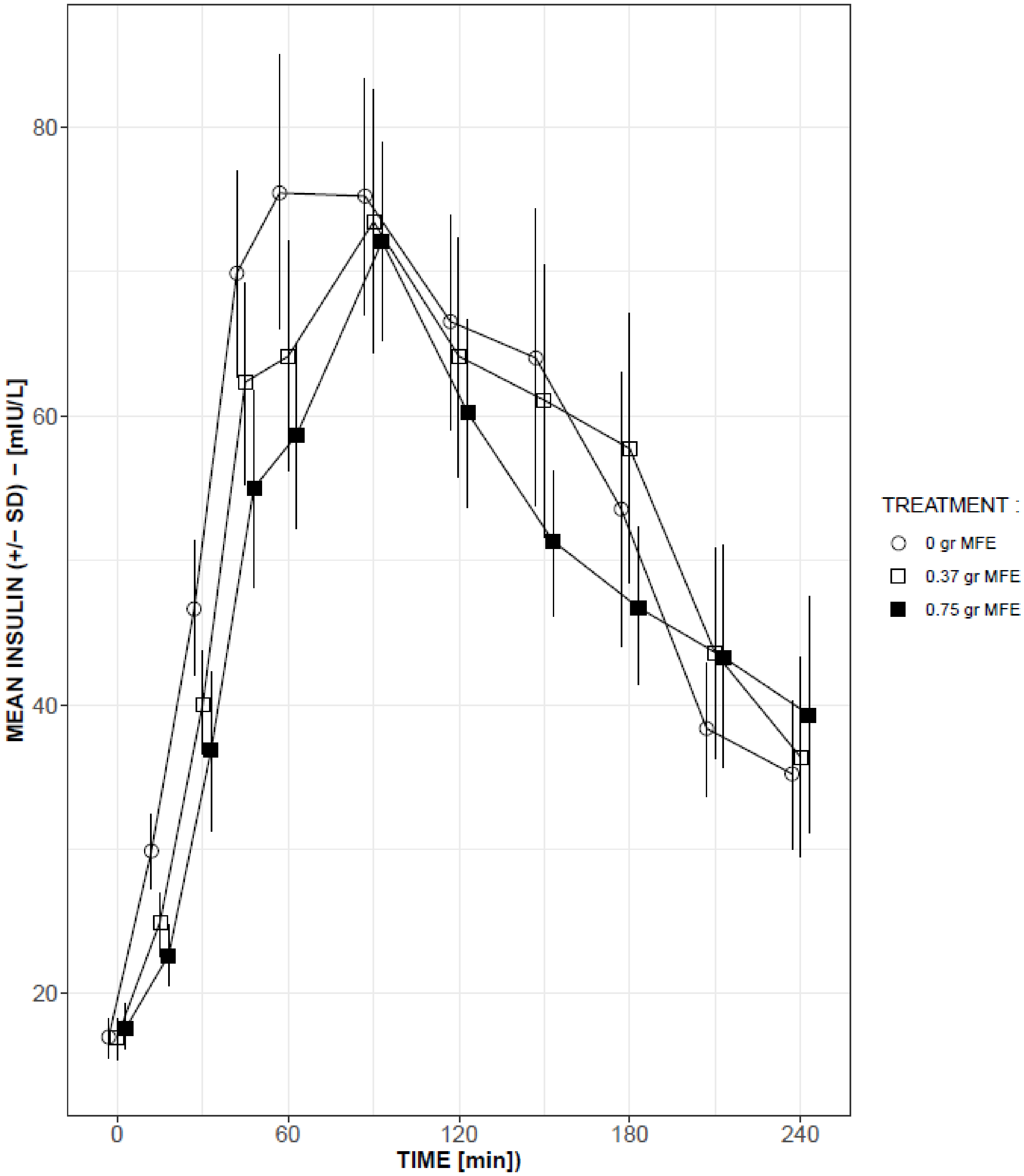

Table S2. Plasma glucose response over 3 hours following consumption of mulberry fruit extract (MFE) added to boiled rice.

Intervention	N	Mean glucose +iAUC$_{3hr}$ (lower, upper 95% CI), min·mmol/L	Mean % difference, MFE vs control (lower, upper 95% CI)
Control	21	448 (380, 529)	
Control + 0.37 g MFE	22	454 (396, 521)	1.3 (-13.0, 18.0)
Control + 0.75 g MFE	20	385 (307, 485)	-14.1 (-32.5, 9.2)

Table S3. Plasma glucose response over 4 hours following consumption of mulberry fruit extract (MFE) added to boiled rice.

Intervention	N	Mean glucose +iAUC$_{4hr}$ (lower, upper 95% CI), min·mmol/L	Mean % difference, MFE vs control (lower, upper 95% CI)
Control	21	487 (406, 583)	
Control + 0.37 g MFE	22	511 (442, 591)	4.9 (-10.5, 23.1)
Control + 0.75 g MFE	20	440 (349, 555)	-9.6 (-29.0, 9.8)

Table S4. Serum insulin response over 3 hours following consumption of mulberry fruit extract (MFE) added to boiled rice.

Intervention	N	Mean insulin tAUC$_{3hr}$ (lower, upper 95% CI), min·mIU/L	Mean % difference, MFE vs control (lower, upper 95% CI)
Control	21	9625 (8155, 11358)	
Control + 0.37 g MFE	22	9040 (7670, 10654	-6.1 (-15.4, 4.2)
Control + 0.75 g MFE	20	8009 (6782, 9460)	-16.8 (-25.2, -7.4)

Table S5. Serum insulin response over 4 hours following consumption of mulberry fruit extract (MFE) added to boiled rice.

Intervention	N	Mean insulin $tAUC_{4hr}$ (lower, upper 95% CI), min·mIU/L	Mean % difference, MFE vs control (lower, upper 95% CI)
Control	21	11763 (10023, 13805)	
Control + 0.37 g MFE	22	11339 (9672, 13294)	-3.6 (-11.9, 5.5)
Control + 0.75 g MFE	20	10175 (8665, 11949)	-13.5 (-21.1, -5.1)

Table S6. Maximum glucose level (C_{max}) over 4 hours following consumption of mulberry fruit extract (MFE) added to boiled rice.

Intervention	N	Mean Cmax (lower, upper 95% CI), mmol/L	Mean % difference, MFE vs control (lower, upper 95% CI)
Control	21	11.5 (10.9, 12.1)	
Control + 0.37 g MFE	22	10.9 (10,4, 11.5)	-4.9 (-10.2, 0.6)
Control + 0.75 g MFE	20	10.7 (10.1, 11.2)	-7.3 (-12.5, -1.7)

Table S7. Glucose swing (C_{max}-C_{min}) over 4 hours following consumption of mulberry fruit extract (MFE) added to boiled rice.

Intervention	N	Mean glucose swing (lower, upper 95% CI), mmol/L	Mean difference, MFE vs control (lower, upper 95% CI), mmol/L
Control	21	4.6 (3.9, 5.3)	
Control + 0.37 g MFE	22	3.8 (3.2, 4.5)	-0.8 (-1.4, -0.2)
Control + 0.75 g MFE	20	3.6 (2.9, 4.2)	-1.1 (-1.7, -0.4)

Table S8. Pooled urine glucose concentration over 4 hours following consumption of mulberry fruit extract (MFE) added to boiled rice, change vs baseline.

Intervention	N	Mean urine glucose at baseline (lower, upper 95% CI), µg/ml	Mean pooled urine glucose post-meal (lower, upper 95% CI), µg/ml	Change in mean pooled urine glucose post-pre meal (lower, upper 95% CI), µg/ml	Mean difference in change from control (lower, upper 95% CI), µg/ml
Control	22	4.0 (2.5, 5.5)	24.4 (15.5, 33.3)	20.4 (11.9-28.9)	
Control + 0.37 g MFE	22	4.3 (3.8, 4.8)	23.5 (21.5, 25.5)	19.2 (15.2, 23.1)	-1.2 (-9.2, 6.8)
Control + 0.75 g MFE	20	4.2 (2.7, 5.7)	24.9 (16.2, 33.6)	20.7 (12.1, 33.2)	0.3 (-11.7, 12.0)
